# The Edge of Perinatal Viability: Understanding the Dutch Position

**DOI:** 10.3389/fped.2021.634290

**Published:** 2021-02-01

**Authors:** L. De Proost, E. J. T. Verweij, H. Ismaili M'hamdi, I. K. M. Reiss, E. A. P. Steegers, R. Geurtzen, A. A. E. Verhagen

**Affiliations:** ^1^Department of Medical Ethics, Philosophy and History of Medicine, Rotterdam, Netherlands; ^2^Department of Neonatology, Rotterdam, Netherlands; ^3^Department of Obstetrics and Gynecology, Rotterdam, Netherlands; ^4^Department of Obstetrics, Leiden University Medical Center (LUMC), Leiden, Netherlands; ^5^Department of Neonatology, Radboud University Medical Center, Amalia Children's Hospital, Nijmegen, Netherlands; ^6^Department of Pediatrics, University Medical Center Groningen, University of Groningen, Groningen, Netherlands

**Keywords:** extremely premature birth, threshold of viability, guidelines, decision-making, medical ethics

## Abstract

The current Dutch guideline on care at the edge of perinatal viability advises to consider initiation of active care to infants born from 24 weeks of gestational age on. This, only after extensive counseling of and shared decision-making with the parents of the yet unborn infant. Compared to most other European guidelines on this matter, the Dutch guideline may be thought to stand out for its relatively high age threshold of initiating active care, its gray zone spanning weeks 24 and 25 in which active management is determined by parental discretion, and a slight reluctance to provide active care in case of extreme prematurity. In this article, we explore the Dutch position more thoroughly. First, we briefly look at the previous and current Dutch guidelines. Second, we position them within the Dutch socio-cultural context. We focus on the Dutch prioritization of individual freedom, the abortion law and the perinatal threshold of viability, and a culturally embedded aversion of suffering. Lastly, we explore two possible adaptations of the Dutch guideline; i.e., to only lower the age threshold to consider the initiation of active care, or to change the type of guideline.

## Introduction

Guidelines on care at the edge of perinatal viability differ between countries. Both in terms of such guidelines and the related attitudes of healthcare professionals, the Netherlands can be considered as an outlier. Our country has a relatively high threshold of providing active care (>24 weeks of gestation), a gray zone between 24 and 26 weeks of gestational age (GA), the initiation of active management in the gray zone determined by parental discretion, and a slight reluctance to initiate active care for extremely premature infants (1–4). This Dutch position merits reflection, especially in view of the current revision of the guideline on the matter. Our article proceeds as follows. First, we provide a concise overview of the Dutch guidelines. Then, we situate the guidelines within the context of Dutch socio-cultural norms and values. Third, we will use the outcomes of this analysis to speculate on possible emendations of the current guideline.

## The History of Dutch Guidelines On Treatment at the Edge of Viability

Until 2005, a Dutch consensus guideline recommended not to provide active care to extremely premature infants born before 26^0/7^ weeks of GA (5). A revised guideline endorsed by the Netherlands Association of Pediatrics and the Netherlands Association of Obstetrics and Gynecology was published in 2005. This guideline recommended the provision of active care to infants born at 25^0/7^ weeks GA and older (6). Both guidelines were strictly GA-based and left room for parental discretion: a management plan was always to be made by the healthcare team together with the parents. In the following years, it was found that the care approach for extremely premature infants was not uniform among medical centers in the Netherlands (7). Moreover, compared to other European countries, perinatal mortality for extremely premature infants in the Netherlands was found to be high (8). In response to these findings, in 2008 the Minister of Health, Welfare and Sport in the Netherlands asked the Netherlands Organization for Scientific Research—Medical Sciences to develop a new guideline on the postnatal management of extremely premature infants. The objective was to harmonize treatment and care at the edge of viability in all Dutch perinatal and neonatal centers (7).

This “new” guideline, published in 2010 under the title *Perinataal beleid bij extreme vroeggeboorte* (Perinatal policy for extreme prematurity) is, again, strictly GA-based (7). The guideline accounts for spontaneous premature births only; thus, does not account for iatrogenic premature births. One of the recommendations states that, after prenatal counseling, providing active care is an option for infants from 24^0/7^ weeks GA onwards, *unless* prognostic factors clearly suggest otherwise. Importantly, the period spanning the 24th and 25th weeks GA is seen as a gray zone characterized by prognostic uncertainty. The management of infants born in this gray zone should be decided on the basis of a consensus between the healthcare professionals and the parents, provided the latter have been extensively counseled and the principles of shared decision-making have been adhered to (9). It should be noted that other countries more often identify this gray zone as a period somewhere between 22 and 24 weeks GA (10). Another guideline recommendation is that extremely premature infants from 23^4/7^ weeks GA should be transferred to a specialized perinatology center—where the best possible care and parental counseling can be provided. Antenatal corticosteroids are recommended to be administered from a GA of 23^5/7^ weeks. Lastly, a cesarean is to be considered from 24^0/7^ weeks GA, balancing both maternal and fetal risks as the consequence for future pregnancies (Table 1).

**Table 1:** GA thresholds in recommendations in Dutch guidelines.

	**Intra-uterine referral**	**Antenatal steroids**	**C-section**	**Resuscitation**
<2005	≥26^0/7^	≥26^0/7^	≥26^0/7^	≥26^0/7^
2005	≥25^0/7^^*^	≥25^0/7^^*^	≥25^0/7^	≥25^0/7^
^*^Gray area between 24^0/7^*and24*^6/7^: *consult a tertiary center*				
2010	≥23^4/7^	≥23^5/7^	≥24^0/7^	≥24^0/7^

Unfortunately, since 2010 only few studies on Dutch management of extreme prematurity have been conducted that provide insights in the effects of the new recommendations. In 2017, the first follow-up results after implementation of the guideline were published (11). The results concern extremely premature infants at the corrected age of 2 years: of those born at 24 weeks, 20% had mild disabilities, 20% had more severe disabilities, and 60% had no disabilities at all. In comparison, 71% of the infants born at 25 weeks had no disabilities at all. Another study shows that in 2011, infants born at 24 weeks had a 43% chance to survive, while infants born at 25 weeks had a 61% chance to survive. Of those born at 24 weeks, 79% had short-term morbidities such as bronchopulmonary dysplasia and retinopathy of prematurity, while this was the case for 71% of the infants born at 25 weeks (12). In 2016, Geurtzen et al. reported that the new recommendations succeeded relatively well to harmonize physician preferences concerning the lower threshold of providing active care. However, preferences concerning the upper threshold for offering comfort care still greatly diverge, as well as practices such as offering a cesarean section and providing cardiopulmonary resuscitation (13). Finally, a recent study by van Beek et al. shows that the implementation of the 2010 guideline “resulted in increased neonatal intensive care unit admission rates and postnatal survival” (14). Although these results are useful to reflect on the guideline, more follow-up research on longer term outcomes is required.

## The Dutch Context

### Multidisciplinary and Evidence-Based Guidelines for Centralized Care

To better understand the Dutch guidelines, it is important to look at how they came about. Three factors are key. First, the aim was to have evidence-based guidelines that relied on Dutch national data. Second, the Dutch guidelines were meant to be multidisciplinarily constituted national consensus guidelines. Third, the guidelines were designed to reflect a national and not a local perspective: the approach to care was centralized. The aim was to ensure the streamlined provision of quality perinatal care in the nine level III and level IV centers in different regions in the Netherlands. As both the complex obstetric and the neonatal intensive care are concentrated in these specialized centers, care is rather well organized and coordinated.

### Culture of Freedom and Responsibility

Historically, Dutch culture has been marked by a prioritization of individual freedom and responsibility (15). Currently, the Netherlands is known for its great variety of liberal policies concerning different aspects of life; for example, the use of soft drugs is tolerated, and prostitution is legalized and regulated (16). The Dutch also take a liberal stance in most of the widely debated bioethical dilemmas. In the Netherlands, euthanasia is legalized since 2001 for people who unbearably suffer mentally or physically (17). Euthanasia is also legalized for people who suffer from dementia, and, since 2004, for severely ill newborns (the so-called Groningen Protocol) (18, 19). Currently under debate is the legalization of euthanasia for people who are “tired of living,” people who feel their “life is completed,” and children from 1 to 12 years old who suffer unbearably (20–22). Furthermore, the Netherlands was one of the first countries in the world to legalize abortion in 1984. Currently, abortion is legalized up to 24 weeks of GA (23).

The bioethical policies discussed above might seem to contrast with the Dutch guideline on care at the edge of viability. Compared to countries such as Sweden, Japan, or Canada, which provide active care to babies born at 22 weeks (24–26), the Netherlands can be described as a late adopter: it is advised to only provide active care to infants born from 24 weeks on, after extensive counseling and a process of shared decision-making. This apparent discrepancy might be explained by the Dutch context.

### Aversion to Suffering and Importance of Quality of Life

Surprisingly, the Dutch bioethical policies have a common goal that might explain, rather than conflict with, the caution of the Dutch guideline concerning treatment of extremely premature newborns. Consider the following three Dutch policies: (a) abortion is legalized up to a GA of 24 weeks (23) (b) active care is only provided to extremely premature infants born from 24 weeks on since the prognosis and or expected quality of life is not deemed hopeful below this threshold (7) (c) euthanasia is legalized for people who unbearably suffer physically or mentally and for severely ill newborns with a prognosis of severe future suffering (17, 19). It could be hypothesized that these policies have a similar goal: assuring that people do not *have* to suffer or do not *have* to live with a poor quality of life. We do not claim that these policies have been enacted for these reasons, but they do factually imply a decrease in people who are suffering or (deemed to) have a poor quality of life. Although a country wanting to avoid suffering might not in itself be remarkable, trying to structurally regulate it by laws and guidelines is.

It is instructive to look at the three policies against the background of the Dutch valuation of freedom and responsibility. The options to abort, euthanize, or to offer comfort care to a child with a poor or infaust prognosis are examples of ways in which persons can exercise their freedom over their life or the life of their offspring. The corollary of such freedom is an emphasis on the responsibility to exercise it in the way one sees fit. Dutch bioethical policies thus provide the possibility to minimize suffering as well as the possibility to exercise one's freedom. Freedom, responsibility, and the avoidance and alleviation of suffering might, in that sense, be interrelated.

## The Future of the Dutch Guideline

As the 2010 guideline is currently under revision, it is interesting to speculate on possible changes. Let us suppose, for the sake of argument, that the guideline *will* change. It seems that there are two main ways that it could. The first is to continue with a strictly GA-based guideline, but with another threshold to consider the initiation of active care. This change would be in line with those of previous revisions and would better align the Dutch guideline with those in other countries. The second way is to change the type of guideline. It could become a more personalized or prognosis-based guideline, advising to take into account other factors than solely GA. But first we discuss two general challenges for revising the Dutch guideline.

### The Threshold of Viability

A first challenge relates to the threshold of viability. Consider again the Dutch abortion policy. The Dutch abortion law is based upon the threshold of viability. In turn, the meaning of this threshold is determined in the Dutch criminal law: Article 82a states that, “Taking the life of a person or of an infant at birth or shortly afterwards shall include: the killing of a fetus which might reasonably be expected to have the potential to survive outside the mother's body” (27). Once the fetus is, in that sense, viable, abortion is illegal, and the acting physician is punishable for murder. Obviously, the guideline on care for extremely premature infants also interrelates with the threshold of viability. Importantly, it can be asked whether lowering the threshold of viability in the guideline for extreme prematurity would demand a similar change in the threshold for legal abortion. This question merits more reflection, as it seems illogical for a country to apply a threshold of viability of 24 weeks in Law X, and at the same time claim in Guideline Y that babies from 23 weeks can survive. One way to avoid such inconsistency would be to find another basis for abortion law than the threshold of viability. Some European countries do not base abortion law on a threshold of viability but refer to a certain GA: in Belgium and Germany, for example, abortion is legalized up to 12 weeks GA (28, 29), in Sweden abortion is legalized up to 18 weeks GA (30). It could be questioned whether changing the abortion law would find support: the Dutch have mostly shown great support for their abortion policy, and the support even seems to have increased over time among the younger generation (31).

Generally, a revision of the guideline on care at the edge of viability must include a reflection on the concept of a threshold of viability. The biological threshold of viability is as yet unknown. One could wonder how much technological interference is “allowed” to still label a certain GA as threshold of viability. In practice, a set GA is actually always an estimated (e-)GA (32). Moreover, because of differences between countries in availability of technological and medical support, thresholds of viability may differ from country to country (7, 24). Of note, countries which have more resources and better infrastructure often also seem to have a lower threshold of viability (33). This raises questions of fairness and equity. All in all, the concept requires reflection. A threshold of viability is a difficult concept to base a law or guideline on. More research on this is urgently required.

### Scarcity of Dutch National Data

Another challenge for revising the 2010 guidelines is the scarcity of Dutch national data about survival, morbidity, and long-term outcomes of extremely premature infants. Except for the current EPI-DAF study, whose results are yet unpublished, Dutch long-term outcomes of extreme prematurity are pending (34). However, even if there *would* be enough national data, the self-fulfilling prophecy of a strict GA-based guideline implies that in the Netherlands, every infant born at 22 or 23 weeks will not have received active care and thus will not have survived (35). Of course, as is seen in other countries, lowering the threshold of viability is a learning curve: it demands time to get used to provide active care to such young infants: results will become better and better in time (36). Moreover, outcome data will always have to be interpreted against a national context and will always be value-loaded: the meaning of concepts such as “surviving,” “quality of life,” “suffering,” and “disability” might differ per country. In the Dutch context, aversion to suffering might color such concepts. Even with enough resources, a culture that structurally avoids suffering might refuse to utilize all technological possibilities: “It is not because we can, that we have to.” Moreover, since the same outcomes can have different meanings in different countries, it is hard to rely on international research to construct national guidelines (37). Taking the difficulties associated with a threshold of viability and the scarcity of national data into account, let us briefly consider the two most plausible options for changing the 2010 guideline.

### Lowering the Threshold in a Strictly GA-Based Guideline

The first option to change the guideline would be lowering the threshold to consider the initiation of active care to 23 or even 22 weeks GA. This would better align the Dutch guidelines with guidelines in other countries and be a similar revision to the ones in 2005 and 2010. Moreover, opting once more for a strictly GA-based guideline would make it easy to use because of its clarity and the limited room for interpretation. The problem is that changing the threshold alone can be considered an outdated measure. There is a lot of current literature stating that GA is not sufficient to come to a prognosis, and that GA is always an estimated (e-)GA (32, 38, 39). Besides, by lowering the threshold to provide active care, the ethical challenges of a strictly GA-based guideline will merely be shifted, not solved.

### Changing the Type of the Guideline

A second option is to change the type of guideline. The decision to provide active care could be broadened to other significant prognostic factors: birth weight, the administration of antenatal corticosteroids, sex, fetal anomalies, and so on. Currently, a trend toward personalizing care at the limits of viability is becoming visible: variation in parents' values and preferences is increasing, and so does the need for a “customization” in care (9, 40). Nevertheless, this option would imply a care approach that is less uniform. This is a downside, given that the main objective of the previous guideline was uniformity. Furthermore, the literature does not provide clear answers yet on how to actually “personalize” in clinical practice around the threshold of viability. Also, evidence on results of and attitudes about these sorts of guidelines is missing. An example of a more personalized type of guideline is that from the United Kingdom, published in 2019 (41). The UK guideline states that neonatal decisions should be based on all relevant prognostic factors and “the best available evidence about the prognosis for the individual baby.” Results concerning the implementation and results of this guideline are not yet available. Nonetheless, opting for a personalized approach cannot avoid the challenges of the threshold of viability as mentioned above. Personalization means that some infants born at 22 weeks will receive active care, and some infants born at 26 weeks will not. If the infants born at 22 weeks survive, this raises once again the question of lowering the threshold of viability.

A guideline on care at the edge of viability must suit the Dutch cultural context, which will in turn increase support from all the stakeholders. Sufficient societal support for change is important as well. It is not just the attitudes of the public and healthcare professionals that are significant. Change should also be feasible in view of the national economic situation, the healthcare system, and the infrastructure of the nine specialized level III and level IV centers. Although one could also argue the opposite: a new guideline might imply a need for more resources, better infrastructure and or more specialized education for healthcare professionals to counsel parents according to those new guidelines. Moreover, mind that not only resources are needed to enable good quality care in the neonatal period but also care that extends into childhood and beyond. Whatever the result of the revision will be, the process should ideally include reflection on the threshold of viability, national interpretation of data, Dutch culture, the societal support base for change, and the availability of resources, infrastructure, and education.

## Conclusion

Some concluding remarks are in order. First, in all its rather exceptional positions in complex bioethical dilemmas, the Netherlands seems to stay true to its own socio-cultural context. The importance of freedom and the wish to structurally avoid suffering coincide in most of its laws and guidelines. Second, the Dutch bioethical landscape stands in need of reflection on the threshold of viability: the interrelation of the abortion law and the guideline for extreme prematurity comes with serious challenges in this regard. Third, a change in the Dutch guideline concerning care at the edge of viability is likely to go one of two ways. In both ways, the new guideline would change the threshold to provide active care, either strictly GA-based or it would opt for a more personalized, prognosis-based approach. Both ways are challenging and require serious reflection.

## Data Availability Statement

The original contributions presented in the study are included in the article/supplementary material, further inquiries can be directed to the corresponding author/s.

## Author Contributions

AAEV, EJTV, and LD have made substantial contributions to the conception of the work. LD wrote a first outline and a draft of the article. This draft was commented on by EJTV and AAEV. Other versions of the article were revised by RG, HI, IKMR, and EAPS. All authors have approved the final version.

## Conflict of Interest

The authors declare that the research was conducted in the absence of any commercial or financial relationships that could be construed as a potential conflict of interest.
